# IgG2a-formatted 4-1BB agonism combined with S100A9 inhibition enhances T cell activation and tumor control in a preclinical model of multiple myeloma

**DOI:** 10.1186/s13046-026-03716-4

**Published:** 2026-05-04

**Authors:** Hatice Satilmis, Adrien Denis, Emma Verheye, Arne Van der Vreken, Dewen Zhan, Evan Calliauw, Marie Törngren, Helena Eriksson, Sylvia Faict, Elke De Bruyne, Eline Menu, Karin Vanderkerken, Kim De Veirman

**Affiliations:** 1 Translational Oncology Research Center, Lab of Hematology and Immunology, Vrije Universiteit Brussel, Laarbeeklaan 103, Brussels, 1090 Belgium; 2 Active Biotech AB, Lund, Sweden; 3 Translational Oncology Research Center, Team Hematology and Immunology, UZ Brussel, Laarbeeklaan 101, Brussels, 1090 Belgium

**Keywords:** Multiple myeloma, Immunotherapy, 4-1BB agonist, Tasquinimod, Immune microenvironment

## Abstract

**Background:**

Immunotherapy has emerged as a promising strategy for multiple myeloma (MM), yet relapse remains frequent due to the immunosuppressive bone marrow (BM) microenvironment, characterized by T cell dysfunction and accumulation of immunosuppressive myeloid cells. The co-stimulatory receptor 4-1BB (CD137, TNFRSF9) can enhance T and NK cell effector functions, but its therapeutic utility in MM is not well established. Tasquinimod (TQ), a clinical-stage S100A9 inhibitor, offers a complementary approach by limiting the recruitment and activity of suppressive myeloid cells.

**Methods:**

4-1BB expression was assessed during disease progression in MM mice and in newly diagnosed MM patients using single-cell RNA sequencing and flow cytometry. Therapeutic potential was evaluated in 5TGM1 tumor-bearing mice treated with two 4-1BB agonists, LOB12.3 (IgG1κ) and 3H3 (IgG2a), using isotype controls. The lead agonist was subsequently combined with TQ to investigate dual targeting of the immunosuppressive tumor microenvironment. Tumor burden was quantified via BM and spleen plasmacytosis and serum M-protein levels. Immune modulation was analyzed using multi-parameter flow cytometry. Statistical significance was determined using the Mann–Whitney U test or one-way ANOVA (*p* < 0.05).

**Results:**

4-1BB expression progressively increased on T and NK cells during tumor development in mice. In primary MM patient BM samples, ex vivo 4-1BB stimulation with urelumab enhanced effector responses, increasing IFN-γ^+^ and Granzyme B^+^ CD3^+^ T cells, alongside trends toward increased CD56^+^ NK cells and elevated IFN-γ^+^ NK cell activity.

In vivo, 4-1BB agonist treatment promoted expansion of T cell subsets, with clone-specific effects: the IgG2a clone 3H3 significantly reduced M-protein levels and BM plasmacytosis, whereas the IgG1 clone LOB12.3 induced NK cell depletion and demonstrated limited anti-tumor activity. Combining 3H3 with TQ provided superior anti-myeloma efficacy, reducing BM plasmacytosis from 62.5% in controls to 14.1% under combination treatment. Mechanistically, the combination enhanced Granzyme B expression, effector T cell differentiation, and dendritic cell maturation (CD86 upregulation), collectively overcoming BM immunosuppression.

**Conclusions:**

These findings establish the isotype-specific efficacy of 4-1BB agonists and support 4-1BB stimulation combined with TQ as a promising strategy to enhance durable immunotherapeutic responses in MM.

**Graphical Abstract:**

**Figure:**
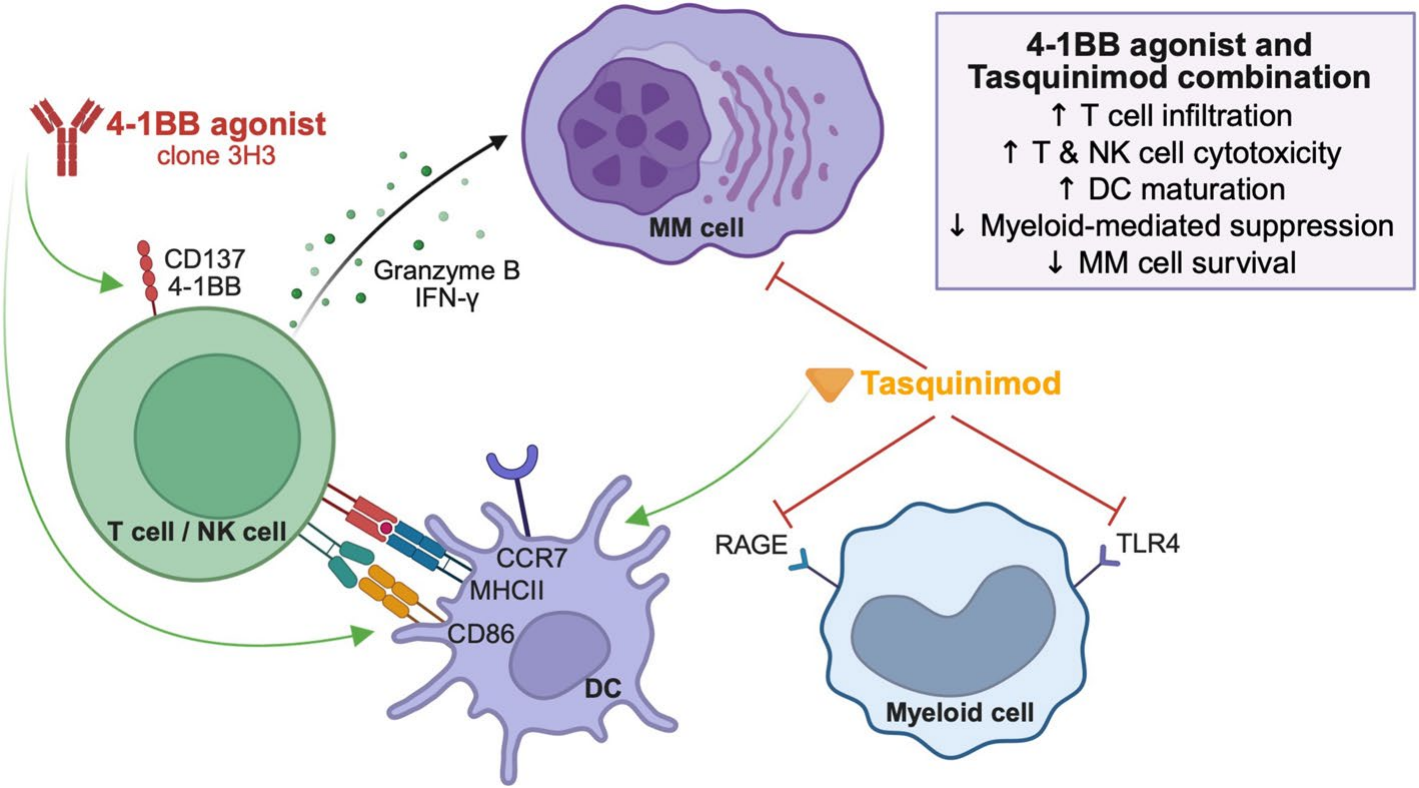

**Supplementary Information:**

The online version contains supplementary material available at 10.1186/s13046-026-03716-4.

## Introduction

Multiple myeloma (MM) is a malignant disorder of terminally differentiated plasma cells that accumulate in the bone marrow (BM), leading to end-organ damage and immune dysregulation. Despite major therapeutic advances with proteasome inhibitors, immunomodulatory drugs, monoclonal antibodies, and cellular therapies, MM remains incurable, and most patients eventually relapse [1–3]. This underscores the need for novel immunotherapeutic strategies that can elicit potent, durable anti-tumor immunity.

Durable anti-cancer immunity relies on effective T cell co-stimulation, making targeted modulation of co-stimulatory pathways a promising therapeutic strategy. Among the co-stimulatory receptors, 4-1BB (CD137, TNFRSF9) is a member of the tumor necrosis factor receptor superfamily, transiently expressed on activated CD8^+^ and CD4^+^ T cells, natural killer (NK) cells, NKT cells, and certain dendritic cell (DC) subsets [4–6]. Engagement of 4-1BB by its ligand (4-1BBL) or agonistic antibodies delivers strong survival and proliferative signals, enhances effector cytokine production, and promotes the generation of long-lived memory T cells. In addition, 4-1BB reinvigorates tumor-experienced T cells, fostering their clonogenic expansion [7–10]. These properties have made 4-1BB an attractive target for cancer immunotherapy.

Preclinical studies have demonstrated that 4-1BB agonists can reverse T cell dysfunction, augment cytotoxicity, and improve tumor control in both solid tumors and hematologic malignancies [11–14]. However, first-generation clinical agents, such as the agonistic antibodies urelumab and utomilumab, showed limited efficacy as monotherapies and were associated with dose-limiting toxicities, particularly hepatotoxicity [14, 15]. These findings highlight the need for careful agonist selection, dosing optimization, and rational combination strategies to maximize benefit while minimizing toxicity.

The BM microenvironment in MM presents unique challenges, including enrichment of immunosuppressive myeloid cells, regulatory T cells (Tregs), and tumor-associated macrophages (TAMs), all of which contribute to impaired T and NK cell responses [16–18]. Since 4-1BB is expressed on both cytotoxic and regulatory immune populations, and can also modulate antigen-presenting cell function, targeted 4-1BB stimulation offers the potential to rebalance immune dynamics within this niche. Furthermore, combining 4-1BB agonism with agents that counteract myeloid immunosuppression (e.g., tasquinimod) or inhibitory checkpoint blockade (e.g., CTLA-4, PD-1/PD-L1 inhibitors) may produce synergistic effects [19–22].

In this study, we performed a comprehensive preclinical and translational evaluation of 4-1BB in MM, using murine 5TMM models and primary patient samples. We mapped 4-1BB expression across immune compartments during disease progression, directly compared the therapeutic performance of two distinct agonist clones, and assessed the benefit of combining 4-1BB stimulation with the myeloid-targeting agent tasquinimod. Our findings provide mechanistic insight into 4-1BB biology in MM and identify a combination approach that enhances its therapeutic potential.

## Methods

### Mice and tumor model

Female C57BL/KaLwRij mice, aged six to eight weeks, were purchased from Envigo (Horst, The Netherlands). All housing conditions, experimental procedures, and follow-up complied with the guidelines of the Belgian Council for Laboratory Animal Science and were approved by the Ethical Committee for Animal Experiments of the Vrije Universiteit Brussel (ECD: 23–281–17; LA1230281).

The 5TGM1 MM model was established by intravenous injection of 1.0 × 10^6^ 5TGM1-eGFP^+^ cells into C57BL/KaLwRij mice. Prior to inoculation, 5TGM1-eGFP^+^ cells were tested for mycoplasma contamination (MycoAlert™, #LT07-418, Lonza, USA) and passaged no more than 1 month prior to experiments.

### Compounds and in vivo experiments

Mice injected with 5TGM1-eGFP^+^ cells were randomly assigned to treatment groups. Treatment started 3 days post-inoculation and continued until animals exhibited signs of morbidity, such as hind-limb paralysis, at which point they were sacrificed. Tasquinimod, provided by Active Biotech AB (Lund, Sweden), was dissolved in 1 N NaOH, and the pH was adjusted to 7.8–8.2. The compound was administered orally to mice via drinking water at a dose of 30 mg/kg, with the solution refreshed twice weekly. The 4-1BB (CD137) agonistic antibodies, clone LOB12.3 (#BE0169) and clone 3H3 (#BE0239), as well as the IgG1κ isotype control (clone HRPN, #BE0088) and IgG2a isotype control (clone 2A3, #BE0089), were obtained from Bio X Cell (Lebanon, New Hampshire, USA). Antibodies and their respective isotype controls were administered intraperitoneally at a dose of 100 µg in a volume of 100 µL, twice per week. Mice were weighed three times per week and monitored for general health throughout the experiment.

### Murine blood and tissue collection

Blood samples (500 µL) were collected from mice via retro-orbital bleeding using Micro Sample Tubes with Serum Gel (Sarstedt AG & Co, Germany). BM cells were isolated by flushing the femurs and tibias with RPMI-1640 medium using a syringe. Spleens were mechanically dissociated by gently pressing them with the back of a sterile syringe to generate single-cell suspensions. BM and spleen suspensions were filtered through a sterile 70 µm cell strainer to remove debris. Red blood cells were lysed, and viable cells were counted using Trypan Blue.

### Tumor burden assessment in mouse model

Tumor burden was evaluated using three complementary methodologies. First, plasmacytosis within BM and spleen samples was quantified by cytospin preparation followed by May-Grünwald Giemsa staining. Briefly, 1 × 10^5^ cells were resuspended in 200 µL RPMI-1640 medium (Thermo Fisher Scientific, Aalst, Belgium) supplemented with 10% heat-inactivated fetal calf serum (FCS; PAN Biotech, Aidenbach, Germany) and centrifuged onto borosilicate glass slides (VWR International, Pennsylvania, USA) at 72 × g for 7 min. Stained cytospins were examined by manual counting of plasma cells in three randomly selected fields. Assessments were performed blinded to avoid bias. Digital images of the stained slides were acquired using a Leica Aperio GT 450 scanner and analyzed with QuPath software (version 0.4.3). Second, serum M-protein levels were determined by serum protein electrophoresis, conducted and analyzed at the clinical laboratory of University Hospital of Brussels (UZ Brussels). The detection limit of this method is approximately 0.5–1.0 g/L. Third, tumor load in BM and spleen was quantified by flow cytometry based on eGFP expression in 5TGM1 cells.

### Ex vivo analysis of primary MM patient samples

Ethical approval and informed consent for BM aspirates in MM patients was obtained from the Medical Ethical Committee of the university hospital (UZ) Brussel (B.U.N. 143201838414). All samples were anonymized by the UZ Brussel biobank. Ethical approval and informed consent for BM aspirates of MM patients at the Department of Medicine and Surgery of the University of Parma were obtained by its Institutional Review Boards and were anonymized in accordance with the Declaration of Helsinki.

Mononuclear cells were isolated from BM samples by density gradient centrifugation using Lymphoprep™ (STEMCELL Technologies, Grenoble, France). For ex vivo assays, mononuclear cells were seeded at a concentration of 5 × 10^5^ cells/mL in 24-well plates in RPMI-1640 medium supplemented with 10% FCS, 2 mM L-glutamine, 1% penicillin/streptomycin, 1% sodium pyruvate, and 1% MEM non-essential amino acids (Thermo Fisher Scientific). Cells were treated with 20 µg/mL of mouse IgG4 isotype control antibody (clone R1, Selleck Chemicals, Houston, Texas, USA), 20 µg/mL of α4-1BB antibody (934823–49–1, Selleck Chemicals), 25 µM of tasquinimod (Sigma-Aldrich, Diegem, Belgium), or a combination of α4-1BB antibody and tasquinimod. Tumor burden and immune cell composition were assessed by flow cytometry after 72 h of treatment.

### Immune compartment assessment by flow cytometry

Samples were washed with phosphate-buffered saline (PBS) and stained with Fixable Viability Dye eFluor™ 506 (1:1000 dilution; eBioscience) for 30 min at 4 °C. To block non-specific antibody binding to Fc receptors, cells were incubated with FcR-blocking reagent (1:200 dilution in PBS; Miltenyi Biotec) for 10 min at 4 °C. Subsequently, cells were stained with a surface antibody mixture diluted in MACS buffer supplemented with 2 mM ethylenediaminetetraacetic acid (EDTA) and 0.5% FCS and incubated for 20 min at 4 °C. Following surface staining, cells were fixed using either the eBioscience™ Intracellular Fixation & Permeabilization Buffer Set (Thermo Fisher Scientific, catalog #88–8824–00) or the eBioscience™ Foxp3/Transcription Factor Staining Buffer Set (Thermo Fisher Scientific, catalog #00–5523–00) according to the manufacturer’s instructions. Intracellular staining was performed by incubating cells with the respective antibody mix diluted in the provided wash buffer for 20 min at 4 °C for all markers, except for Foxp3, IFN-γ, and granzyme B, which required overnight incubation at 4 °C. For optimal detection of intracellular cytokines, including IFN-γ and Granzyme B in NK and T cells, murine single-cell suspensions were pre-stimulated for 4 h at 37 °C with 5% CO_2_ in RPMI-1640 medium supplemented with 10% FCS, 300 µg/mL L-glutamine, 100 U/mL penicillin, 100 µg/mL streptomycin, 1 mM non-essential amino acids, 1 mM sodium pyruvate, 0.02 mM 2-mercaptoethanol, 5 µg/mL Brefeldin A (BioLegend), 20 ng/mL phorbol 12-myristate 13-acetate (PMA; Sigma-Aldrich), and 1 µg/mL ionomycin (Invitrogen). Antibodies used in this study are listed in Suppl. Table 1 for mouse experiments and Suppl. Table 2 for human samples.

To assess the tumor burden upon therapy in primary human BM samples, cells were stained with 7’aminoactinomycin D (7-AAD) (BD Biosciences) and CD138-PE (clone: REA929, Miltenyi Biotec). Flow cytometry data were acquired on a BD LSRFortessa (BD Biosciences) and analyzed using FlowJo software (version 10.8.1; Ashland, Oregon, USA). Marker expression was quantified using mean fluorescence intensity (MFI), and ΔMFI (delta MFI) was calculated as the MFI of the stained sample minus the MFI of the corresponding fluorescence minus one (FMO) control. ΔMFI was used to indicate relative changes in marker expression between experimental and control populations.

### Single-cell RNA-sequencing datasets

The murine single-cell RNA-sequencing dataset (GSE271306) generated by our group was derived from the 5T33MM mouse model at defined stages of disease progression. BM and spleen samples were collected at three timepoints: naïve (healthy control), 14 days post-inoculation (DPI; 14DPI), and end-stage disease, defined by the onset of paralysis (*n* = *4* for each condition, pooled). CD45^+^ idiotype^-^/CS1^-^ 7-AAD^-^ viable cells were isolated by fluorescence-activated cell sorting (FACS) and subjected to single-cell transcriptomic profiling using the Chromium Controller platform (10 × Genomics). Library preparation, sequencing, and downstream computational analysis were performed as previously described [23].

For the human analysis, publicly available single-cell RNA-sequencing datasets (GSE124310 and GSE161801) were reanalyzed and integrated by our group. These datasets include CD138^-^CD45^+^ sorted immune cell populations from primary BM samples of healthy donors (*n* = *9*), patients diagnosed with monoclonal gammopathy of undetermined significance (MGUS, *n* = *5*), smoldering MM (SMM, *n* = *11*) and newly diagnosed MM (NDMM, *n* = *7*) [23]. Graphs were generated using Cell Ranger v.6.1.2, R v.4.2.3, Seurat v.4.0.5 and v.5.0.1.

### Software

All schematic illustrations were created using BioRender.com.

### Statistical analysis

For *in vivo* experiments, the sample size was calculated using G*Power. Statistical analyses and data visualization were performed using GraphPad Prism 10.2.3 software. For pairwise comparisons, the one-tailed unpaired Mann–Whitney U-test was used. For multiple group comparisons, the Kruskal–Wallis test or one-way ANOVA test was used. All data represent the mean ± standard deviation (SD). *p < 0.05; **p < 0.01; ***p < 0.001; ****p < 0.0001 were considered statistically significant.

## Results

### 4-1BB is a co-stimulatory marker expressed on T and NK cells in MM-bearing mice

Immunotherapeutic strategies targeting costimulatory pathways have gained increasing interest recently, with 4-1BB emerging as a promising target. To characterize 4-1BB expression across different immune cell populations in MM-bearing mice, we analyzed a previously generated scRNA-seq dataset from the 5T33MM model, in which 17 distinct immune cell populations were identified and UMAP-clustered in both BM and spleen [23]. 4-1BB gene (*Tnfrsf9*) expression was detectable in both organs.

**Fig. 1:**
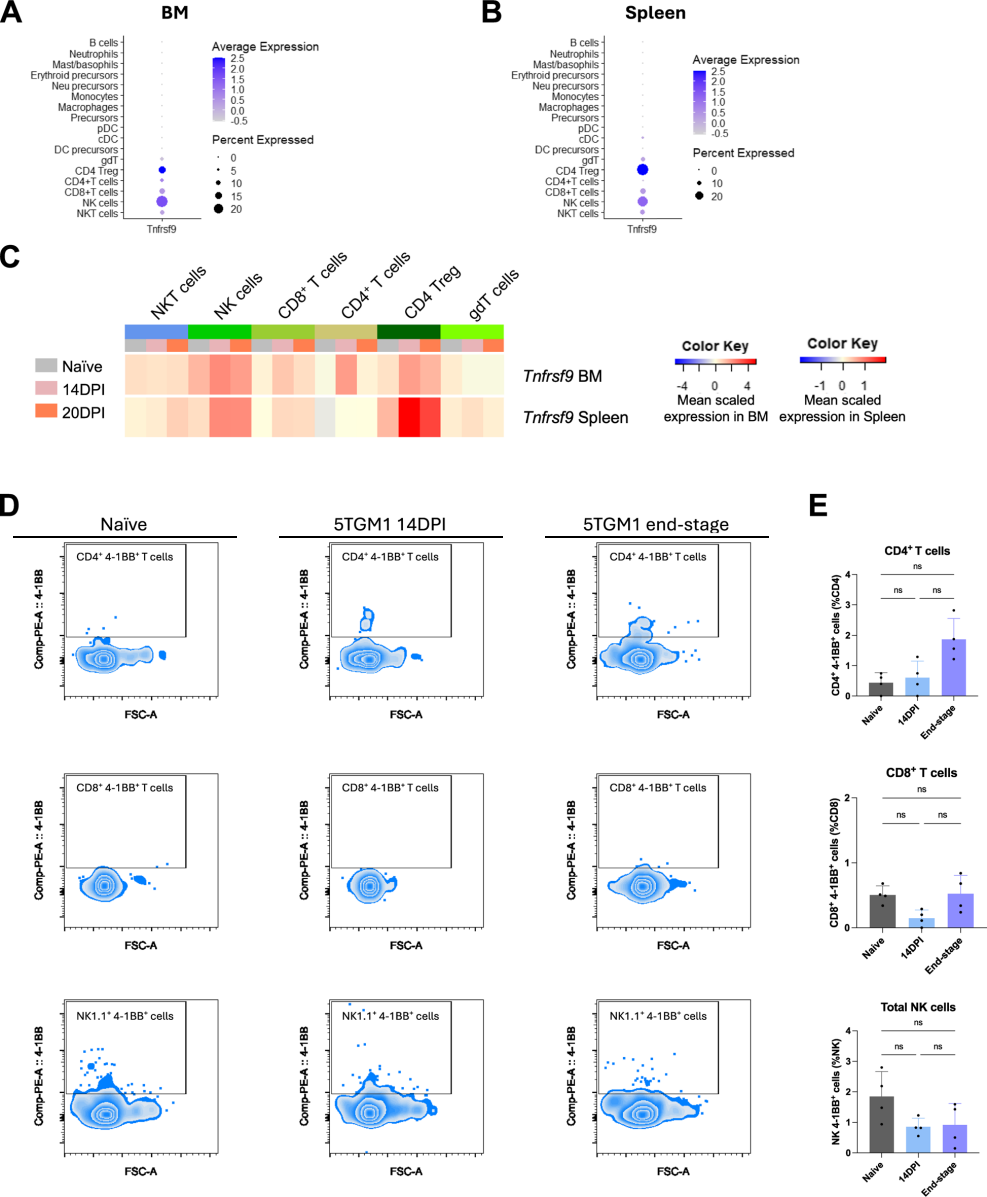
4-1BB is a therapeutically targetable co-stimulatory marker on T and NK Cells in MM-bearing mice. **A**-**B** Dot plots showing the expression of murine 4-1BB gene *Tnfrsf9* in BM (**A**) and spleen (**B**) within the murine 5T33 scRNA-seq dataset. Dot size represents the percentage of cells expressing the gene and color gradient represents the average scaled expression within each cell cluster. **C** Heatmap showing the expression of 4-1BB gene *Tnfrsf9* in BM and spleen across different disease stages in the murine 5T33 scRNA-seq dataset. **D** Representative flow cytometry plots showing the frequencies of 4-1BB^+^ in CD4^+^ T, CD8^+^ T cells and NK cells in BM across different disease stages. **E** Bar graphs showing the relative abundance of 4-1BB^+^ CD4^+^ T, CD8^+^ T cells and NK cells, as determined by flow cytometry. *n* = 4 per group, statistical analysis was performed by Kruskal–Wallis test: ns: not significant, error bars indicate SD

In the BM, *Tnfrsf9* was expressed across multiple lymphocyte subsets, with the highest levels observed in Tregs and NK cells. Moderate expression was detected in CD8^+^ T cells, CD4^+^ T cells, and NKT cells. As MM progressed, expression levels in the BM increased significantly, reaching peak levels at day 14 post-inoculation (DPI14), particularly in CD4^+^ T cells, CD8^+^ T cells, Tregs, and NK cells (Fig. 1A-C).

In the spleen, the expression pattern was similar. *Tnfrsf9* was most prominently expressed in Tregs and NK cells, while CD4^+^ T cells showed minimal expression. MM progression was associated with upregulation of *Tnfrsf9* in splenic Tregs and NK cells (Fig. 1B-C). However, expression levels in the spleen were lower compared to the BM, as shown by the scale in Fig. 1C.

These findings were validated using flow cytometry in the 5TGM1 MM model, which is a moderate and slow progressive model, widely employed in immunomodulatory drug studies. Analysis revealed that 4-1BB expression on CD4^+^ T cells peaked at end-stage disease, whereas NK cells exhibited the highest 4-1BB expression in the naïve mice (Fig. 1D-E). Consistent with the transcriptional data, BM samples showed higher levels of 4-1BB expression compared to the spleen (Suppl. Fig. 1A-B).

### Differential antitumor activity of 4-1BB agonist clones LOB12.3 and 3H3 in the 5TGM1 MM model

Two 4-1BB agonist monoclonal antibodies, urelumab (IgG4) and utomilumab (IgG2), have been previously evaluated in clinical trials for a range of solid tumors and hematologic malignancies [13, 24–26], underscoring the therapeutic potential of 4-1BB targeting to enhance T cell-mediated anti-tumor immunity. Building on this rationale, we examined the activity of two commercially available 4-1BB agonist clones in a preclinical model of MM. We selected LOB12.3 (IgG1κ) and 3H3 (IgG2a) for their established preclinical use and well-characterized functional properties, which allow the study of FcγR-dependent mechanisms, such as antibody-dependent cellular cytotoxicity and immune cell depletion, that can influence in vivo efficacy [27]. This design provides mechanistic insight into how antibody isotype and Fc-engagement affect anti-tumor activity, complementing clinical studies using IgG4 and IgG2 antibodies.

**Fig. 2:**
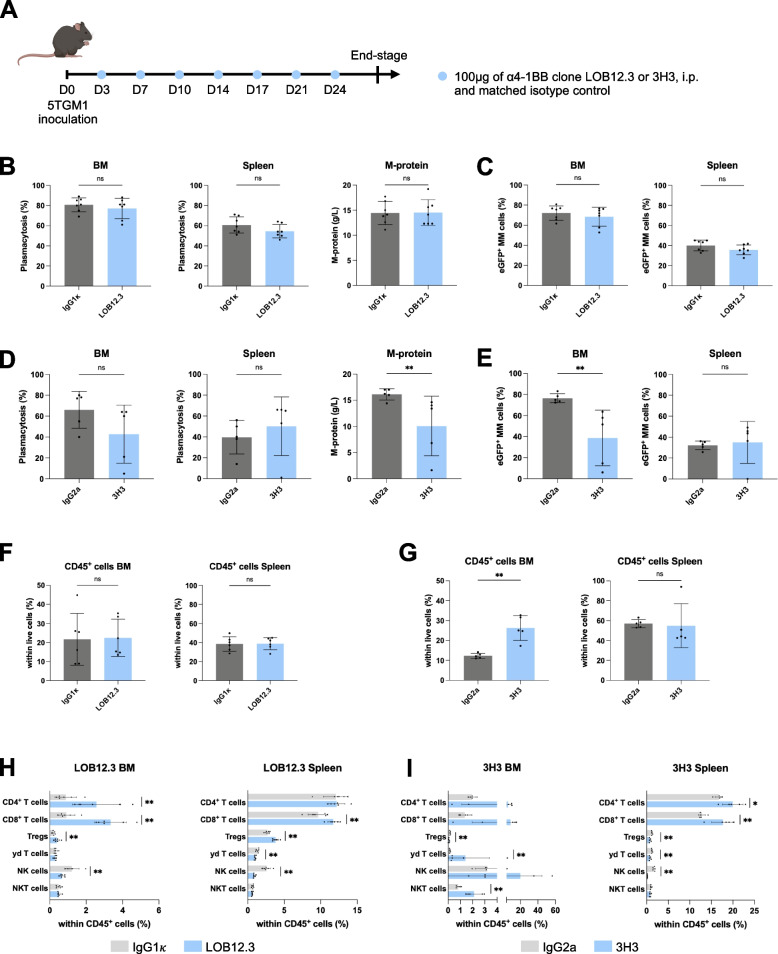
Differential antitumor activity of 4-1BB agonists clones LOB12.3 and 3H3 in 5TGM1 MM model. **A** Mice were inoculated intravenously with 5TGM1 MM cells and treated intraperitoneally starting on day 3 post-inoculation with 100μg of either α4-1BB agonistic antibody clone LOB12.3 or clone 3H3, alongside their respective matched isotype controls (IgG1κ for LOB12.3 and IgG2a for 3H3). Mice were sacrificed at disease end-stage. **B** Tumor burden was evaluated by assessing plasmacytosis in BM and spleen using May-Grünwald Giemsa–stained cytospin and quantifying serum M-protein levels, following treatment with α4-1BB clone LOB12.3. **C** Bar graphs show the relative abundance of eGFP^+^ 5TGM1 MM cells, as determined by flow cytometry, following treatment with α4-1BB clone LOB12.3. *n* = 7 per group. **D** Tumor burden following treatment with α4-1BB clone 3H3 was evaluated by assessing plasmacytosis in BM and spleen using May-Grünwald Giemsa–stained cytospin and quantifying serum M-protein levels. **E** Bar graphs show the relative abundance of eGFP^+^ 5TGM1 MM cells, as determined by flow cytometry, following treatment with α4-1BB clone 3H3. *n* = 5 per group. **F**-**G** Total CD45^+^ frequency within the live cells compartment in BM and spleen, determined by flow cytometry, following treatment with α4-1BB clone LOB12.3 (**F**) and clone 3H3 (**G**). **H**-**I** T cell and NK cell subsets within the CD45^+^ compartment in BM and spleen, analyzed by flow cytometry following treatment with LOB12.3 (**H**) or 3H3 (**I**) *n* = 6 and *n* = 5 per group, respectively. Statistical analysis was performed by Mann–Whitney U test: *p < 0.05, **p < 0.01, error bars indicate SD

Immunocompetent mice were inoculated with eGFP^+^ 5TGM1 cells and treated twice weekly with 100 μg of either 4-1BB agonist clone LOB12.3, clone 3H3 or its matched isotype controls (Fig. 2A). Throughout the treatment period, body weight was monitored as a surrogate marker of systemic toxicity, and no significant differences in body weight were observed between treatment and control groups (Suppl. Fig. 2A-B). Tumor burden was assessed at end-stage, defined by the first signs of paralysis in the isotype-treated groups, based on plasmacytosis, serum M-protein (as marker of MM paraprotein), and the percentage of eGFP^+^ malignant plasma cells in BM and spleen via flow cytometry.

Treatment with clone LOB12.3 (*n* = 7) did not significantly reduce tumor burden across all parameters analyzed (Fig. 2B-C). In contrast, mice treated with clone 3H3 (*n* = 5) demonstrated a significant decrease in both serum M-protein levels and in the percentage of eGFP^+^ cells in the BM (mean, 76.4% vs 38.7%), indicating effective tumor control (Fig. 2D-E). No significant differences in tumor burden parameters were observed in the spleen following treatment with either clone 3H3 or LOB12.3. These results indicate that clone 3H3, but not LOB12.3, exhibits in vivo anti-myeloma activity in BM in the 5TGM1 model.

To investigate immunomodulatory effects of both clones, we analyzed immune cell populations by flow cytometry (Fig. 2F-I). Treatment with clone 3H3 resulted in an increased frequency of CD45^+^ cells in the BM (mean, 12.3% vs 26.3%) (Fig. 2G). Within the CD45^+^ compartment, both LOB12.3 and 3H3 showed a trend toward increased frequencies of 4‑1BB–expressing CD4^+^ and CD8^+^ T lymphocytes, although these changes were not statistically significant (Fig. 2I). The two clones showed clearly distinct effects on regulatory and innate lymphocyte subsets: LOB12.3 increased Treg frequencies in both BM and spleen and reduced NK-cell frequencies, whereas 3H3 decreased Treg frequencies in both compartments, with NK-cell reduction observed only in the spleen (Fig. 2H-I).

The differential impact on NK cells across tissues may account for the limited efficacy of 3H3 treatment in reducing splenic tumor burden. To further dissect this phenomenon and determine whether early NK‑cell depletion contributes to the reduced therapeutic effect, we examined the immediate immunological consequences of 4‑1BB stimulation before the onset of pronounced tumor‑driven immunosuppression and tissue remodeling. Using a short‑term treatment window, 5TGM1‑inoculated MM mice were analyzed 10 days post–tumor inoculation, an early phase with minimal disease burden (1–2% eGFP^+^ cells in BM and spleen), after receiving two doses of the 4‑1BB agonist clone 3H3 or its matched isotype control (Suppl. Fig. 3A–B). At this early time point, NK and NKT cell frequencies in the BM remained unchanged following 3H3 treatment (Suppl. Fig. 3C), whereas both populations were markedly diminished in the spleen (Suppl. Fig. 3D). Annexin V/PI staining revealed a significant increase in late apoptotic NK cells specifically within the spleen, with no corresponding increase in the BM, indicating that 4‑1BB stimulation directly induces apoptosis and drives acute NK‑cell loss in peripheral tissues (Suppl. Fig. 3E–F).

Phenotypic profiling further showed that splenic NK cells upregulated the inhibitory receptors TIM-3 and LAG-3, while PD-1 expression remained unchanged at this early time point. In contrast, NK cells in the BM did not exhibit alterations in these markers (Suppl. Fig. 3G-H). Moreover, splenic NK cells showed increased CD25 expression, consistent with heightened activation, alongside a parallel reduction in both CD16^+^ and CD16^-^ NK subsets (Suppl. Fig. 3I–L). The broad loss of CD16 aligns with activation-induced shedding rather than selective depletion of a cytotoxic subset. Additionally, the frequency of CCR5^+^ NK cells decreased (Suppl. Fig. 3K–L); however, the absence of NK accumulation in the BM argues against substantial redistribution. Collectively, these findings demonstrate that early 4-1BB agonism triggers strong activation followed by apoptosis-mediated depletion of splenic NK cells, providing a mechanistic explanation for the reduced local antitumor activity of 3H3 in the spleen.

### 4-1BB is expressed on T and NK cells in MM patients and its targeting enhances immune cell activation

**Fig. 3:**
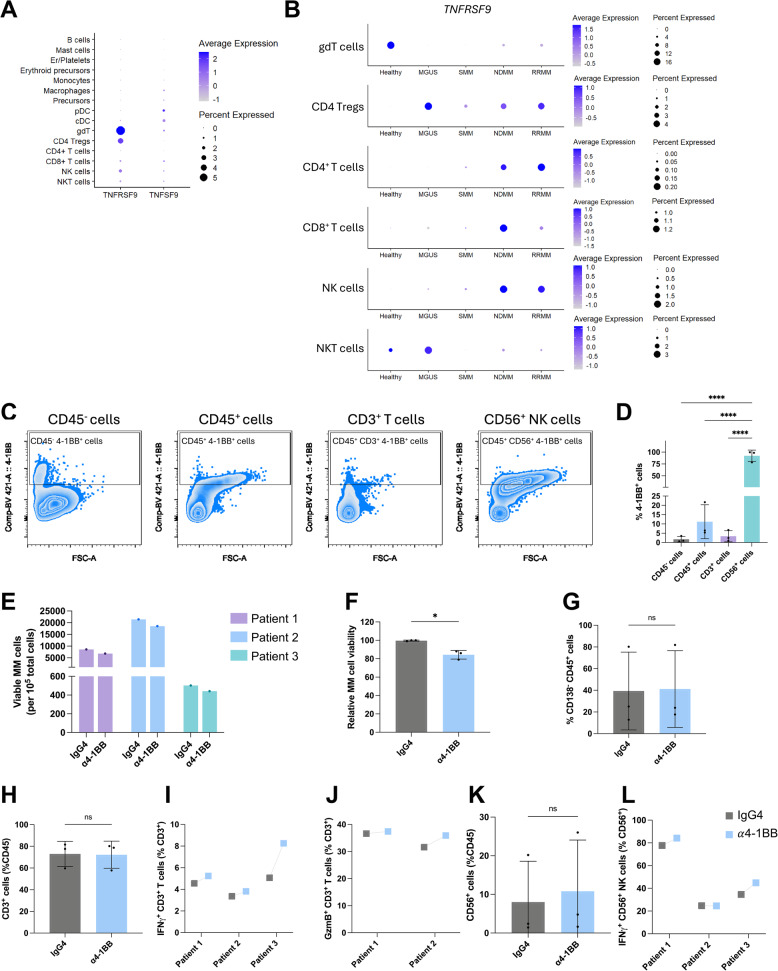
4-1BB is expressed on T and NK cells in MM patients and its targeting enhances immune cell activation. **A** Dot plots showing expression of human 4-1BB gene *TNFRSF9* and its ligand 4-1BBL (*TNFSF9)* in the human BM scRNA-seq dataset. Dot size represents the percentage of cells expressing the gene, and color gradient represents the average scaled expression within each cell cluster. Data from primary BM samples of healthy donors (*n* = *9*), patients diagnosed with monoclonal gammopathy of undetermined significance (MGUS, *n* = *5*), smoldering MM (SMM, *n* = *11*) and newly diagnosed MM (NDMM, *n* = *7).* **B** Dot plots showing expression of human 4-1BB gene *TNFRSF9* in T and NK cell subtypes across different disease stages. **C** Representative flow cytometry plots showing frequencies of 4-1BB^+^ cells in CD45^−^, CD45^+^, CD3^+^ T cells and CD56^+^ NK cells from BM aspirates of MM patients. **D** Bar graph showing the relative abundance of 4-1BB^+^ cells in CD45^−^ cells, CD45^+^ cells, CD3^+^ T cells and CD56^+^ NK cells, as determined by flow cytometry. *n* = 3, statistical analysis was performed by One-way ANOVA: ****p < 0.0001, error bars indicate SD. **E** Bar graph showing viable MM cells per 10^5^ total cells after 72 h ex vivo treatment with isotype control IgG4 or α4-1BB agonistic antibody. **F**-**H** Bar graphs showing relative MM cell viability (**F**), CD138^−^CD45^+^ immune cells (**G**) and CD3^+^ T cells (**H**) after 72 h ex vivo treatment. **I**-**J** Paired scatter plots of IFN-γ (**I**) and Granzyme B (**J**) in T cells after 72 h ex vivo treatment. Patient 3 was not included in panel J due to insufficient cell numbers for reliable analysis. **K**-**L** Bar graph showing CD56^+^ NK cells (**K**) and paired scatter plot of IFN-γ in CD56^+^ NK cells (**L**) after 72 h ex vivo treatment; *n* = 3, statistical analysis was performed by Mann–Whitney U test: *p < 0.05, error bars indicate SD

To directly evaluate the therapeutic relevance of targeting 4-1BB in human MM, we examined 4-1BB (*TNFRSF9*) expression using publicly available scRNA-seq datasets (GSE124310 and GSE161801), which were reanalyzed and integrated by our group. Consistent with our 5T33MM scRNA-seq data, this analysis confirmed low but detectable expression of 4-1BB across several immune subsets, most prominently in γδ T cells, Tregs, and NK cells, with lower expression in CD4^+^ and CD8^+^ T cells (Fig. 3A), supporting the translational relevance of the 5TMM models.

Interestingly, 4-1BB (*TNFRSF9*) gene expression in CD4^+^, CD8^+^, and NK cells increased following MM development, suggesting that disease-driven upregulation of 4-1BB may enhance responsiveness to 4-1BB–targeted immunotherapy (Fig. 3B).

Flow cytometric analysis further validated 4-1BB expression on CD3^+^ T and CD56^+^ NK cells, revealing higher levels on CD45^+^ cells compared with CD45^-^ cells, with CD56^+^ NK cells showing the highest expression (Fig. 3C-D). Ex vivo treatment of newly diagnosed primary patient samples with urelumab for 72 h resulted in a modest reduction in tumor load (Fig. 3E-F), while the frequency of CD45^+^ immune cells remained unchanged (Fig. 3G). Ex vivo 4-1BB stimulation with urelumab increased frequencies of IFN-γ^+^ and Granzyme B^+^ CD3^+^ T cells, while the percentage of CD3^+^ T cells remained stable (Fig. 3H-J). This was accompanied by trends toward a modest increase in CD56^+^ NK cells and elevated IFN-γ^+^ NK cells (Fig. 3K-L). These findings collectively provide translational evidence supporting 4-1BB as a viable immunotherapeutic target in MM.

### Co-targeting T cell and myeloid compartments using α4-1BB and tasquinimod enhances anti-MM activity

**Fig. 4:**
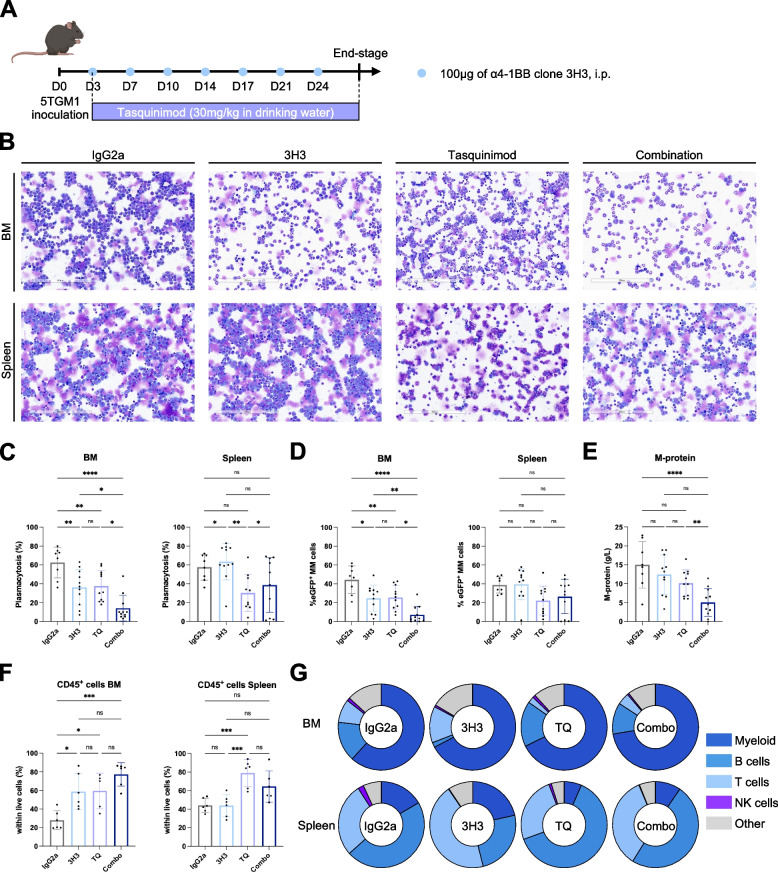
Co-targeting T cell and myeloid compartments using α4-1BB and tasquinimod enhances anti-MM activity. **A** Mice were inoculated with 5TGM1 MM cells and randomized into four treatment groups. Mice were treated intraperitoneally starting on day 3 post-inoculation with 100μg of α4-1BB agonistic antibody clone 3H3, or its matched isotype control (IgG2a). Tasquinimod was administered in drinking water at 30mg/kg, either as monotherapy or in combination with α4-1BB. Mice were sacrificed at disease end-stage. **B** Representative May-Grünwald Giemsa stained cytospins of BM (top) and spleen (bottom) from 5TGM1 MM bearing mice treated with isotype control, α4-1BB, tasquinimod, or the combination therapy. Scale bar: 200μm. **C**-**E** Tumor burden was evaluated by assessing plasmacytosis in BM and spleen using May-Grünwald Giemsa–stained cytosmears (**C**), measuring eGFP^+^ tumor cell infiltration in BM and spleen by flow cytometry (**D**) and quantifying serum M-protein levels (**E**) following treatment. *n* = 8 for IgG2a group and *n* = 11 for all other groups. **F** Total CD45^+^ frequency within the live cells compartment in BM and spleen, analyzed by flow cytometry following treatment. **G** Pie charts showing the relative abundance of myeloid cells (CD11b^+^), B cells (CD19^+^), T cells (TCRβ^+^), NK cells (NK1.1^+^), and other immune cell populations in BM (top) and spleen (bottom), as determined by flow cytometry. *n* = 6 per group. Statistical analysis was performed by One-way ANOVA: ns: *p* ≥ 0.05, *p < 0.05, **p < 0.01, ***p < 0.001, and ****p < 0.0001, error bars indicate SD

While 4-1BB agonists primarily activate tumor-experienced T cells to further enhance anti-tumor immunity, their effectiveness may be limited by the immunosuppressive tumor microenvironment, particularly the presence of suppressive myeloid cells. To address this, we investigated the use of Tasquinimod (TQ), a myeloid-targeting agent currently in phase Ib/IIa clinical trials for relapsed MM. In our previous work, TQ demonstrated efficacy in two preclinical MM models by modulating the immunosuppressive myeloid compartment [28]. By combining 4-1BB agonism with TQ, we aimed to concurrently promote T cell activation and alleviate myeloid-driven immune suppression, thereby enhancing the overall anti-tumor response. MM-bearing mice were treated with TQ, the 4-1BB agonist clone 3H3, or their combination until reaching ethical endpoints (Fig. 4A). Tumor burden was assessed by cytospin-based plasmacytosis scoring, serum M-protein levels, and flow cytometry analysis of eGFP^+^ MM cells. In the BM, both TQ and 4-1BB monotherapies significantly reduced tumor burden, as evidenced by decreased plasmacytosis (Fig. 4B-C) and reduced frequencies of eGFP^+^ MM cells (mean: isotype, 44.4%; 3H3, 24.6%; TQ, 25.6%; combination, 7.1%; Fig. 4D). Notably, 4-1BB agonist treatment alone showed no significant anti-tumor effect in the spleen, consistent with observations from the previous experiment (Fig. 2), whereas TQ monotherapy led to a clear reduction in splenic tumor load (Fig. 4B-D). The combination therapy did not provide additional benefit over TQ monotherapy in the spleen, while in the BM it induced a significant reduction in tumor burden exceeding the effects of either monotherapy. This was accompanied by a significant decrease in serum M-protein levels, reflecting improved systemic tumor control (Fig. 4E).

To gain insights into the immunological response to 4-1BB and TQ treatment and to investigate the observed differences between the BM and spleen, we analyzed the impact of both therapies on immune cell populations using flow cytometry (Fig. 4F-G). In the BM, the percentage of viable CD45^+^ immune cells significantly increased following both TQ and 4-1BB monotherapies (Fig. 4F). Combination therapy led to a substantial increase in the frequency of CD45^+^ cells compared to isotype-treated mice. In the spleen, TQ monotherapy induced a significant increase in CD45^+^ cells, while the combination treatment showed a similar but non-significant upward trend (Fig. 4F).

A detailed analysis of the CD45^+^ immune population revealed that the frequency of CD11b^+^ myeloid cells remained unchanged following combination treatment in both the BM and spleen (Fig. 4G; Suppl. Fig. 4A). CD19^+^ B cells were significantly reduced in both compartments after 4-1BB monotherapy, indicating a sensitivity of this population to 4-1BB-mediated signaling; however, no significant difference in B cell frequency was observed between the control and combination groups (Fig. 4G; Suppl. Fig. 4B).

TCRβ^+^ T cells in the BM showed a trend toward increase following 4-1BB treatment, while splenic TCRβ^+^ T cells were significantly increased by both 4-1BB monotherapy and combination therapy, with the strongest effect observed in the 4-1BB group (Fig. 4G; Suppl. Fig. 4C).

**Fig. 5:**
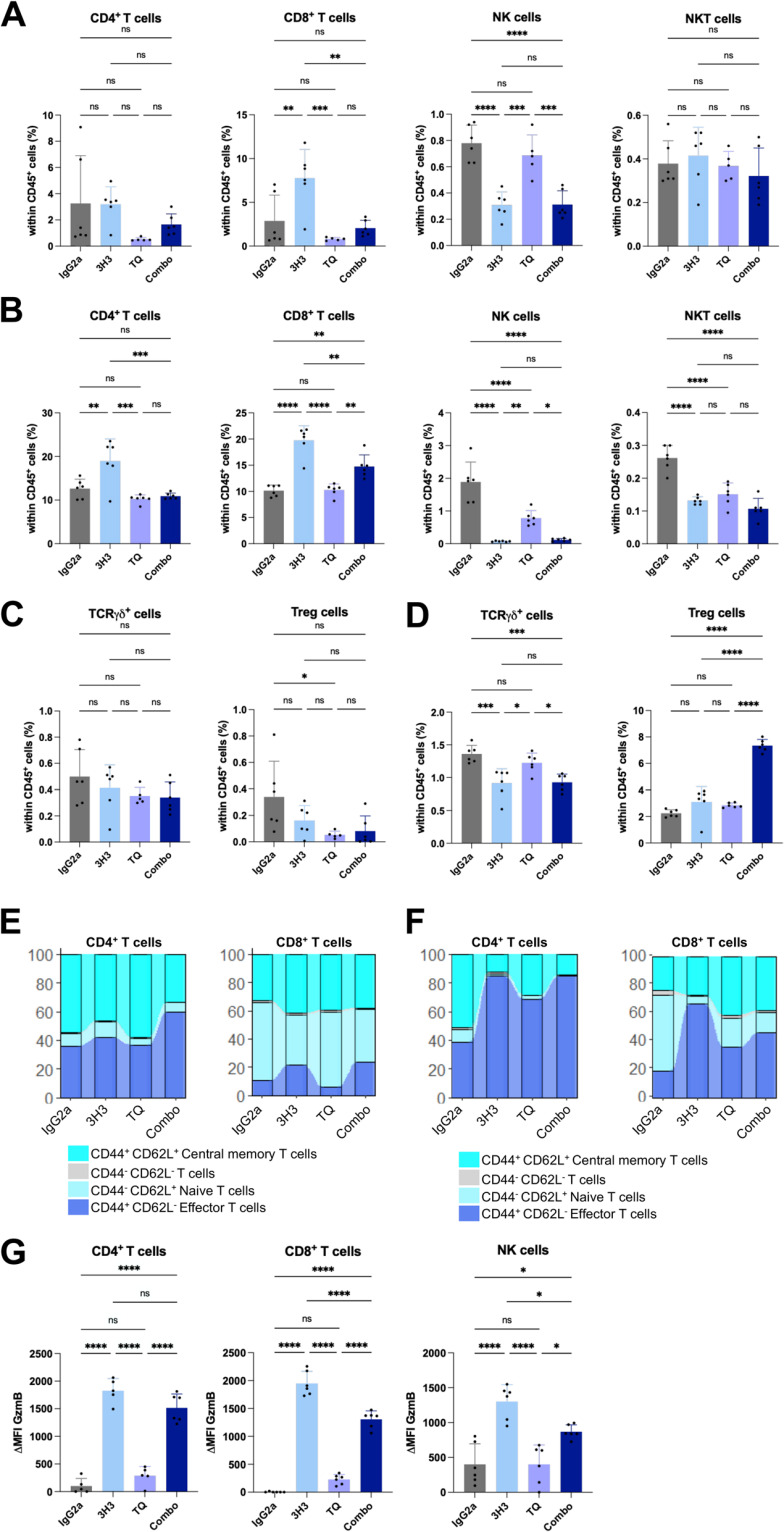
Combination therapy induces Granzyme B–mediated T cell activation and effector differentiation. Mice inoculated with 5TGM1 MM cells were randomized to four treatment groups: α4-1BB clone 3H3, isotype control, tasquinimod, or the combination, after which immune populations were analyzed in both BM and spleen. **A**-**B** Lymphoid subsets within the CD45^+^ compartment in BM (**A**) and spleen (**B**), analyzed by flow cytometry. **C**-**D** γδ T cells (TCRγδ^+^) and regulatory T cells (CD4^+^ Foxp3^+^) within the CD45^+^ compartment in BM (**C**) and spleen (**D**), analyzed by flow cytometry. **E**-**F** Alluvial plots showing the distribution of CD4^+^ and CD8^+^ T cell subsets in BM (**E**) and spleen (**F**), including central memory T cells (CD44^+^CD62L^+^), effector T cells (CD44^+^CD62L^-^), naïve T cells (CD44^-^CD62L^+^), and double-negative T cells (CD44^-^CD62L^-^), analyzed by flow cytometry. **G** ΔMFI of Granzyme B in lymphoid subsets from BM after treatment, analysed using flow cytometry. *n* = 6 per group. Statistical analysis was performed by One-way ANOVA: ns: *p* ≥ 0.05, *p < 0.05, **p < 0.01, ***p < 0.001, and ****p < 0.0001, error bars indicate SD

In addition to modulating T and B cells, 4-1BB-based therapies affected NK cell populations. Both 4-1BB monotherapy and combination therapy caused a significant reduction in total NK cells in the BM (mean: isotype, 0.78%; 3H3, 0.31%; TQ, 0.68%; combination, 0.31%) and near-complete depletion in the spleen (mean: isotype, 1.88%; 3H3, 0.09%; TQ, 0.78%; combination, 0.12%; Figs. 4G and 5A-B). Further investigation revealed that, similar to the short-term treatment setting, NK cells were highly susceptible to depletion, whereas NKT cell frequencies remained stable in the BM and were only partially reduced in the spleen (Fig. 4G, Fig. 5A-B).

Together, these results demonstrate that 4-1BB agonist therapy reduces tumor burden as a monotherapy and achieves superior anti-myeloma activity when combined with the myeloid-targeting agent TQ, as evidenced by enhanced control of disease markers, reduced malignant plasma cell infiltration and induced shifts in the immune microenvironment.

### Combination therapy induces granzyme B–mediated T cell activation and effector differentiation

In BM, a significant increase in BM CD8^+^ T cells was observed following 4-1BB treatment, with no significant change after combination therapy (Fig. 5A). In spleen, CD4^+^ T cells increased significantly after 4-1BB treatment, while CD8^+^ T cells increased in both 4-1BB and combination groups, more markedly with 4-1BB monotherapy (Fig. 5B). Alongside the TCRβ^+^ T cells, 4-1BB agonist therapy led to a significant reduction in splenic TCRγδ^+^ cells, an effect maintained with combination therapy. Tregs exhibited an organ specific pattern: while there was a trend toward decreased frequency following 4-1BB monotherapy and combination therapy in BM, splenic Tregs were significantly increased after combination treatment, indicating a potential modulatory effect of the combined regimen on this immunosuppressive subset (Fig. 5C–D).

Differentiation state analysis showed central memory CD4^+^ T cells significantly decreased in BM and spleen after 4-1BB and combination treatments, whereas central memory CD8^+^ T cells were mostly unchanged (Fig. 5E-F; Suppl. Fig. 5A-B). Regarding effector and naïve subsets, CD4^+^ effector T cells were significantly increased in the BM after combination therapy. In the spleen, all treatment groups showed elevated CD4^+^ effector T cells and a concomitant decrease in naïve CD4^+^ T cells (Suppl. Fig. 5A-B). CD8^+^ effector T cells significantly expanded in both BM and spleen following 4-1BB and combination therapies, accompanied by a significant reduction in naïve CD8^+^ T cells in both compartments (Suppl. Fig. 5A-B).

Functional analyses demonstrated a significant increase in the potency of BM CD4^+^, CD8^+^ T cells, and NK cells to produce Granzyme B following both 4-1BB and combination treatments (Fig. 5G). Splenic CD4^+^ and CD8^+^ T cell Granzyme B levels were unchanged with combination therapy, while splenic NK cells showed decreased Granzyme B expression (Suppl. Fig. 5C).

The early activation marker CD69 was significantly upregulated in BM–derived NK cells following combination therapy, with trends toward increases in BM-derived CD4^+^ and CD8^+^ T cells. In the spleen, CD69 expression was significantly elevated in CD4^+^ T cells (Suppl. Fig. 5D). BM CD4^+^ T cells exhibited significantly enhanced potency to produce IFN-γ following 4-1BB and combination treatments, while in spleen, increased IFN-γ-producing capacity of CD4^+^ and CD8^+^ T cells was observed only with 4-1BB monotherapy (Suppl. Fig. 5E). PD-1 expression showed no significant differences in BM, indicating no evidence of exhaustion; however, splenic PD-1 expression increased significantly after combination therapy (Suppl. Fig. 5F).

Taken together, these findings indicate that 4-1BB stimulation effectively activates T cells, promotes their expansion and differentiation toward an effector phenotype, particularly among CD8^+^ subsets, and enhances functional activity without inducing exhaustion. NK cells, although activated, were markedly depleted. NKT cells appeared more resistant to depletion and maintained activation without signs of exhaustion.

### Combination therapy orchestrates the reshaping of the tumor immune microenvironment

Although 4-1BB stimulation predominantly affects the lymphoid compartment, we next investigated the myeloid populations to explore whether its combination with TQ could reprogram the entire tumor immune microenvironment.

**Fig. 6:**
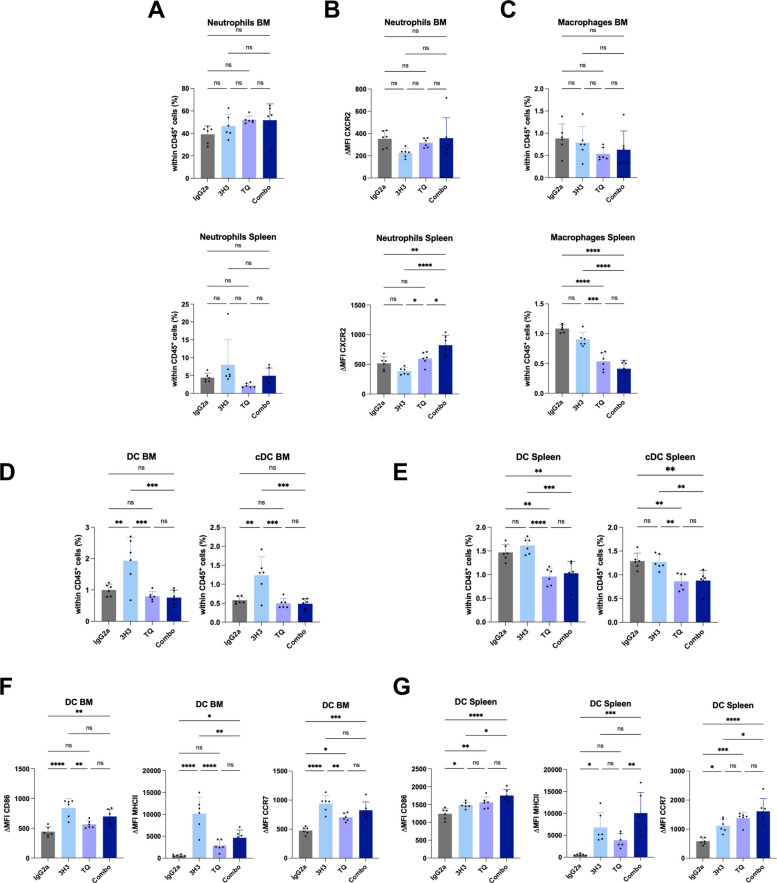
Combination therapy orchestrates the reshaping of the tumor immune microenvironment. **A**-**C** Bar graphs showing the frequencies of neutrophils within the CD45^+^ compartment (CD11b^+^ Ly6G^+^) (**A**), ΔMFI of CXCR2 in neutrophils (**B**) and frequency of macrophages (CD11b^+^ Ly6G^-^ F4/80^+^ MHCII^+^) (**C**) in BM (top) and spleen (bottom). **D**-**E** DC and cDC frequencies within the CD45^+^ compartment in BM (**D**) and spleen (**E**). **F**-**G** ΔMFI of CD86, MHCII, CCR7 in total DCs in BM (**F**) and spleen (**G**) after treatment, analyzed using flow cytometry. *n* = 6 per group. Statistical analysis was performed by One-way ANOVA: ns: *p* ≥ 0.05, *p < 0.05, **p < 0.01, ***p < 0.001, and ****p < 0.0001, error bars indicate SD

As mentioned above, the frequency of CD11b^+^ myeloid cells remained unchanged following combination treatment in both the BM and spleen (Fig. 4G; Suppl. Fig. 4A). Similarly, neutrophil frequencies were unaffected by treatment in either tissue (Fig. 6A); however, splenic neutrophils displayed a pro-migratory and activated phenotype, evidenced by increased CXCR2 expression (Fig. 6B). Other myeloid subsets, including monocytes and eosinophils, were not significantly altered by either monotherapy (Suppl. Fig. 6A-B). In contrast, the percentage of macrophages was significantly reduced in the spleen following both TQ monotherapy and combination therapy, suggesting a specific effect of TQ on this population (Fig. 6C).

In the BM, 4-1BB agonism significantly increased the frequency of total DCs (CD11c^+^ MHCII^+^), driven by an expansion of CCR7^-^ conventional DCs (cDCs), particularly the cDC2 subset, while monocyte-derived DCs (mDCs, CD11c^+^ MHCII^+^ CD64^+^) remained unaffected (Fig. 6D; Suppl. Fig. 6C). In the spleen, 4-1BB treatment selectively expanded cDC1 cells despite an overall reduction in total DC frequency (Fig. 6E; Suppl. Fig. 6D).

To evaluate DC functional activation, we assessed CD86, MHCII and CCR7, key molecules involed in co-stimulation, antigen presentation and migration respectively, essential for T cell priming. Expression levels (ΔMFI) of all assessed markers were significantly upregulated in total DCs in both BM and spleen (Fig. 6F-G). In the BM, CD86 expression was elevated in mDCs following both 4-1BB and combination therapies (Suppl. Fig. 6E). Similarly, cDCs showed significantly increased CD86 levels after 4-1BB treatment (Suppl. Fig. 6E). In the spleen, DC activation was observed across all treatment groups, with the most pronounced CD86 upregulation in the combination therapy group. This increase appeared to arise from both cDC1 and cDC2 subsets (Suppl. Fig. 6F-G).

Analysis of DC activation at an early time point following two doses of a 4-1BB agonist or its isotype control was performed in 5TGM1-inoculated MM mice 10 days post-tumor inoculation. 4-1BB agonism significantly increased the frequency of total DCs in the spleen (Suppl. Fig. 6H-J). CD86 expression was elevated in both BM and spleen, whereas MHCII and CD80, the latter associated with later stages of maturation, remained unchanged. Notably, CCR7 expression was reduced in both tissues, suggesting that DCs had not yet acquired a migratory phenotype or were retained locally during early activation (Suppl. Fig. 6I-K).

Collectively, these results demonstrate that 4-1BB agonist therapy and its combination with TQ engages both adaptive and innate immunity by augmenting T cell cytotoxicity, limiting the recruitment of suppressive myeloid cells and promoting DC activation, thereby fostering a multifaceted anti-tumor immune response.

## Discussion

This study provides a comprehensive preclinical and translational evaluation of 4-1BB (CD137, TNFRSF9) as an immunotherapeutic target in MM, demonstrating that it is broadly expressed across immune subsets, upregulated during disease progression, and can be exploited to enhance anti-myeloma immunity. Using murine 5TMM models and primary human MM samples, we found that the agonist clone 3H3 was capable of inducing robust BM tumor control, while clone LOB12.3 lacked measurable benefit, underscoring the importance of agonist selection for clinical translation. Notably, therapeutic activity was further enhanced by combining 4-1BB stimulation with the myeloid-targeting agent tasquinimod, resulting in superior BM tumor control, augmented T cell cytotoxicity, and enhanced DC activation compared with either monotherapy. These findings highlight the potential of rational combinatorial approaches that simultaneously activate lymphoid effectors and alleviate myeloid-mediated immunosuppression.

Our data extend prior reports of 4-1BB biology in cancer [29–32] by demonstrating disease-driven upregulation not only in CD8^+^ and CD4^+^ T cells but also in NK and Treg populations in both BM and spleen. The BM, a disease-relevant, immunologically distinct compartment, displayed higher 4-1BB expression than the spleen, correlating with the greater therapeutic impact of 3H3 in this tumor site. This organ-specific pattern may reflect differences in immune cell composition, tumor burden, and the immunosuppressive microenvironment, all which shape agonist responsiveness [33].

Functionally, 4-1BB stimulation promoted effector differentiation and cytotoxic molecule production in CD8^+^ T cells without inducing features of exhaustion, consistent with its role as a potent co-stimulatory receptor [34]. Interestingly, NK cells, although activated, were markedly depleted, likely through AICD or heightened sensitivity to sustained 4-1BB signaling, mirroring prior murine findings [31, 35]. Importantly, the observed decrease in NK‑cell frequency following short‑term in vivo 4‑1BB treatment was confirmed to be primarily due to AICD. This effect may be further amplified by FcγR‑mediated mechanisms, given that clone 3H3 is a murine IgG2a antibody with strong FcγR‑engagement capacity. Such Fc‑dependent interactions can promote antibody‑dependent cellular cytotoxicity (ADCC) or fratricide of 4‑1BB–expressing NK cells, providing a mechanistic explanation for the limited efficacy of 4‑1BB monotherapy despite clear evidence of immune activation. In contrast, NKT cells appeared more resistant to depletion, maintaining their functional capacity.

Although 4-1BB is not expressed on DCs, 4-1BB agonism enhanced frequency and activation, as evidenced by increased CD86 expression in both cDC and mDC subsets. This supports a dual mechanism in which 4-1BB directly augments cytotoxic lymphocytes and indirectly sustains T cell priming through professional antigen-presenting cells, likely mediated by damage-associated molecular patterns (DAMPs) and pro-inflammatory cytokines IFN-γ and TNF-α [36, 37]. These indirect effects may also depend on FcγR crosslinking on myeloid cells, which has been shown to critically modulate the agonistic strength of 4-1BB antibodies. 

Although toxicity was not a primary endpoint in this study, prior preclinical work with anti-4-1BB antibodies highlights important safety considerations relevant to clinical translation. In particular, agonistic anti-4-1BB clone 3H3 has been shown to elevate serum alanine aminotransferase (ALT) levels and induce immune cell infiltration in liver tissue in murine models, whereas clones with weaker agonistic activity such as LOB12.3 did not induce comparable hepatic effects. These findings suggest that strong 4-1BB activation, often amplified by FcγR engagement, can be accompanied by hepatotoxicity in vivo, emphasizing the need for detailed biochemical and histopathological evaluation in future studies. Notably, these hepatic effects were not associated with overt morbidity or significant weight loss, and consistent with this, no clinical signs of toxicity were observed under our experimental conditions.

Beyond hepatotoxicity, sustained or systemic 4-1BB agonism also raises the theoretical risk of off-target immune activation or autoimmunity due to broad co-stimulatory signaling across multiple lymphoid subsets. While overt autoimmune manifestations have not been widely reported in murine studies using clone 3H3, dedicated evaluation of autoimmunity-associated pathology and long-term immune dysregulation was outside the scope of the present study and will be essential for future translational development.

Given the central role of immunosuppressive myeloid populations in MM [38, 39], we evaluated the therapeutic impact of combining 4-1BB agonism with tasquinimod, an allosteric S100A9 inhibitor with known activity against suppressive myeloid cells and tumor-associated macrophages [19, 20, 28]. The combination delivered additive benefits: 3H3-driven cytotoxic T cell activation was complemented by tasquinimod-mediated macrophage reduction and neutrophil migration, culminating in superior BM tumor control and reduced systemic disease markers. This cooperative mechanism aligns with the growing recognition that dual targeting of adaptive and innate immunity can overcome microenvironmental resistance in MM. While plasma or tumor concentrations of tasquinimod were not directly measured in this study, continuous administration via drinking water is a well-established approach to maintain pharmacologically active exposure in mice. This dosing strategy is expected to provide consistent target engagement throughout the study period. Nevertheless, the absence of direct pharmacokinetic confirmation in this experiment should be considered when interpreting the results.

The translational relevance of our murine findings was supported by primary human MM samples, where 4-1BB expression was detected across multiple immune subsets and increased during disease progression, consistent with previous observations in MM patient PBMCs [40, 41]. Ex vivo stimulation with urelumab enhanced T and NK cell effector functions and modestly reduced tumor load, reinforcing the clinical feasibility of 4-1BB–based strategies [24–26]. Notably, urelumab is an IgG4 isotype antibody engineered to minimize FcγR engagement and effector functions. In contrast to our murine data obtained with the IgG2a clone 3H3, ex vivo stimulation with urelumab did not significantly affect NK cell frequency, supporting the notion that NK-cell depletion through ADCC is unlikely in humans due to the low FcγR-binding capacity of the IgG4 backbone. While the ex vivo data support the translational relevance of 4‑1BB agonism, these analyses were limited to three patient samples. Validation in larger patient cohorts will be important to further substantiate these findings. Together, these data highlight how antibody isotype, FcγR binding affinity, and pharmacokinetic properties critically influence both therapeutic efficacy and safety, underscoring the importance of Fc engineering in the clinical development of 4-1BB–targeted therapies.

## Conclusions

In conclusion, our work positions 4-1BB as a viable, multifaceted immunotherapeutic target in MM, with efficacy that is enhanced through strategic combination with myeloid-targeting agents such as tasquinimod. Clone selection and antibody isotype emerge as critical determinants of therapeutic success, shaping FcγR engagement, ADCC potential, and toxicity profiles. Looking forward, rational pairing of 4-1BB agonism with other immunotherapies, such as PD-1/PD-L1 checkpoint blockade, together with optimized Fc engineering strategies, may further potentiate anti-myeloma immunity by simultaneously expanding effector T cell pools and preventing exhaustion. Future clinical studies should focus on optimizing combination regimens, elucidating the mechanisms underlying NK cell loss, with particular attention to Fc engineering strategies to enhance therapeutic efficacy, and developing predictive biomarkers to guide patient selection.

## Supplementary Information

Supplementary Material 1.

Supplementary Material 2.

## Data Availability

The datasets analyzed during the current study are available in the GEO repository, under the accession number GSE271306, GSE124310 and GSE161801. Other data are available upon reasonable request.

## Abbreviations

ADCC: Antibody-Dependent Cellular Cytotoxicity
AICD: Activation-Induced Cell Death
ALT: Alanine Aminotransferase
BM: Bone Marrow
cDC: Conventional Dendritic Cell
cDC1: Conventional Dendritic Cell Subtype 1
cDC2: Conventional Dendritic Cell Subtype 2
DAMP: Damage-Associated Molecular Pattern
DC: Dendritic Cell
DPI: Days Post-Inoculation
eGFP: Enhanced Green Fluorescent Protein
FMO: Fluorescence Minus One
mDC: Monocyte-Derived Dendritic Cell
MFI: Mean Fluorescence Intensity
MGUS: Monoclonal Gammopathy of Undetermined Significance
MM: Multiple Myeloma
NK: Natural Killer
NKT: Natural Killer T (Cell)
NDMM: Newly Diagnosed Multiple Myeloma
PD-1: Programmed Cell Death Protein 1
PD-L1: Programmed Death-Ligand 1
scRNA-seq: Single-Cell RNA Sequencing
SMM: Smoldering Multiple Myeloma
TAM: Tumor-Associated Macrophages
TQ: Tasquinimod
Treg: Regulatory T Cell
TNF-α: Tumor Necrosis Factor-Alpha
TNFRSF9: Tumor Necrosis Factor Receptor Superfamily Member 9 (4-1BB)
