# Supplementary material for: IgG2a-formatted 4-1BB agonism combined with S100A9 inhibition enhances T cell activation and tumor control in a preclinical model of multiple myeloma
Source: J Exp Clin Cancer Res. 2026 May 4;45:172. doi: 10.1186/s13046-026-03716-4 (PMC13459263; doi:10.1186/s13046-026-03716-4)
Supplement: Supplementary file 1 [file 13046_2026_3716_MOESM2_ESM.pdf]

Supplementary Information

Supplementary Figures

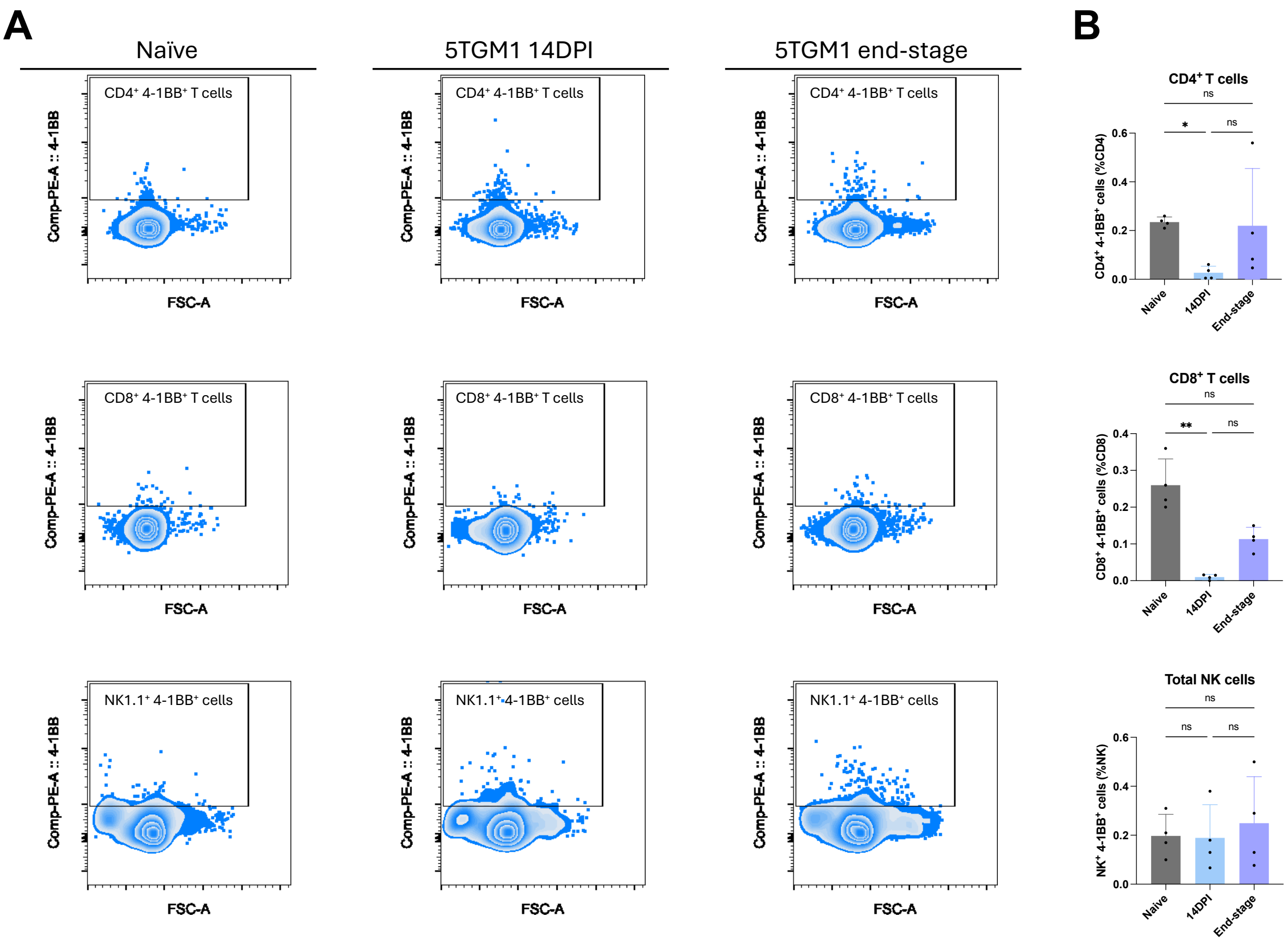

Suppl. Figure 1: 4-1BB expression in splenic immune subsets of MM-bearing mice. (A) Representative flow cytometry plots showing the frequencies of 4-1BB$^+$ in CD4$^+$ T, CD8$^+$ T cells and NK cells in spleen across different disease stages. **(B)** Bar graphs showing the relative abundance of 4-1BB$^+$ CD4$^+$ T, CD8$^+$ T cells and NK cells, as determined by flow cytometry. n=4 per group, statistical analysis was performed by Kruskal-Wallis test: ns: not significant: $p \geq 0.05$, $^*p<0.05$, $^{**}p<0.01$, error bars indicate SD.

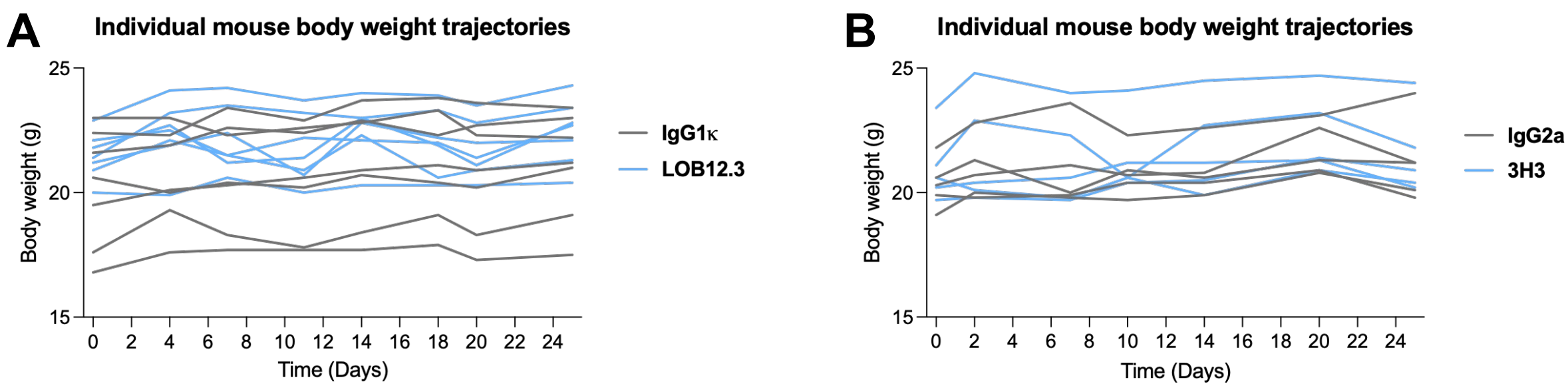

Suppl. Figure 2: Longitudinal body weight monitoring during 4-1BB agonist treatment in the 5TGM1 MM model. Mice were inoculated intravenously with 5TGM1 multiple myeloma (MM) cells and treated intraperitoneally starting on day 3 post-inoculation with 100 µg of the agonistic anti-4-1BB antibodies clone LOB12.3 (A) or clone 3H3 (B), alongside their respective matched isotype controls (IgG1κ for LOB12.3 and IgG2a for 3H3). Body weight was monitored longitudinally throughout the treatment period as an indicator of general health and treatment tolerability. Individual mouse trajectories are shown, with each line representing one mouse.

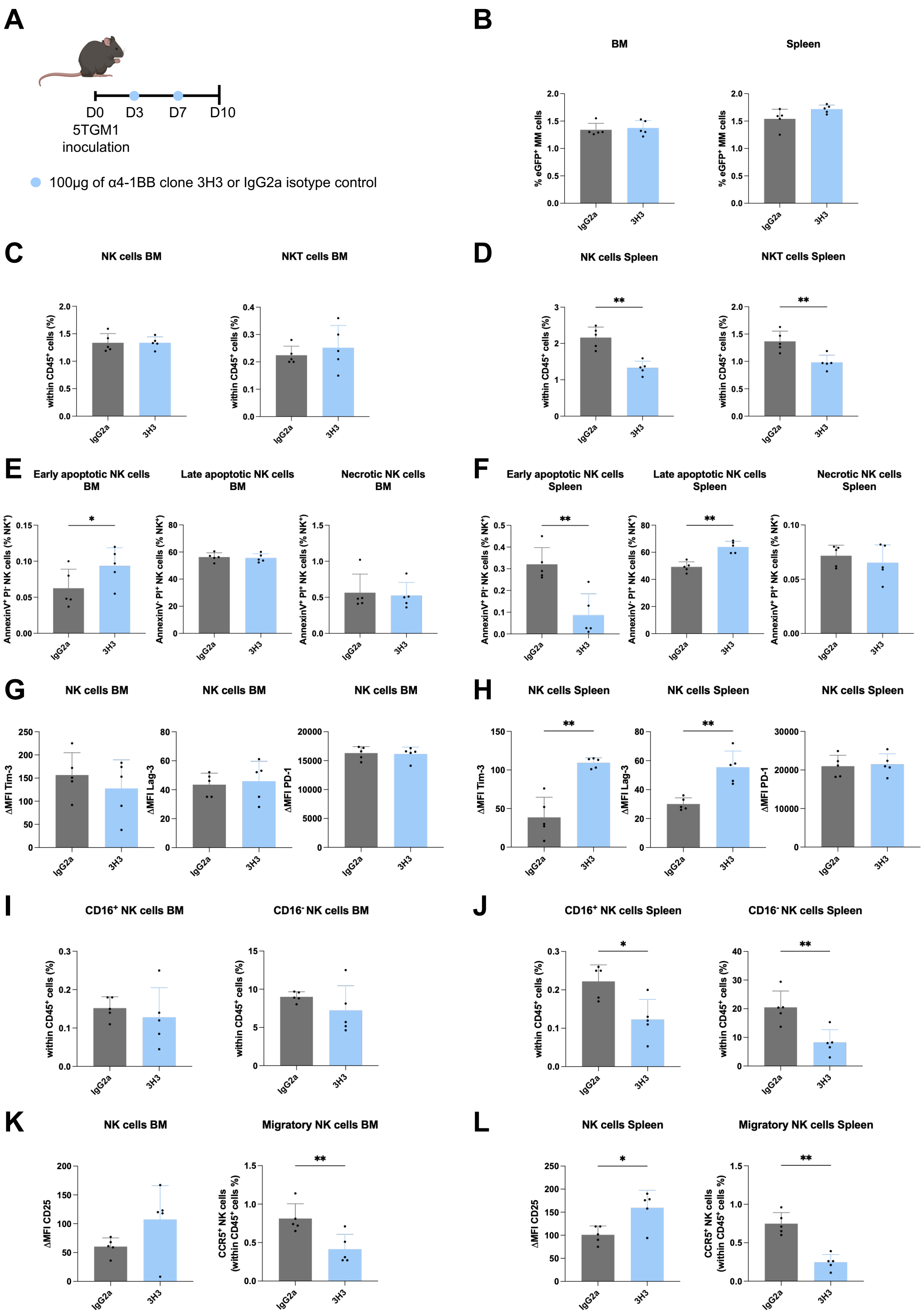
A
D0
D3
D7
D10
5TGM1
inoculation
100µg of α4-1BB clone 3H3 or IgG2a isotype control
B
BM
Spleen
% eGFP⁺ MM cells
C
NK cells BM
NKT cells BM
within CD45⁺ cells (%)
D
NK cells Spleen
NKT cells Spleen
E
Early apoptotic NK cells BM
Late apoptotic NK cells BM
Necrotic NK cells BM
AnnexinV⁺ PI⁻ NK cells (% NK⁺)
AnnexinV⁻ PI⁺ NK cells (% NK⁺)
AnnexinV⁺ PI⁺ NK cells (% NK⁺)
F
Early apoptotic NK cells Spleen
Late apoptotic NK cells Spleen
Necrotic NK cells Spleen
G
NK cells BM
ΔMFI Tim-3
ΔMFI Lag-3
ΔMFI PD-1
H
NK cells Spleen
I
CD16⁺ NK cells BM
CD16⁻ NK cells BM
J
CD16⁺ NK cells Spleen
CD16⁻ NK cells Spleen
K
NK cells BM
Migratory NK cells BM
ΔMFI CD25
CCR5⁺ NK cells (within CD45⁺ cells %)
L
NK cells Spleen
Migratory NK cells Spleen
IgG2a
3H3

Suppl. Figure 3: Short term α4-1BB therapy using clone 3H3 induces apoptosis in NK cells. **(A)** Mice were inoculated intravenously with 5TGM1 MM cells and treated intraperitoneally on day 3 and day 7 post-inoculation with 100μg of α4-1BB agonistic antibody clone 3H3, alongside its matched isotype control IgG2a. Mice were sacrificed at day 10 post-inoculation. **(B)** Bar graphs show the relative abundance of eGFP^{+} 5TGM1 MM cells in BM and spleen, as determined by flow cytometry, following treatment with α4-1BB clone 3H3. **(C-D)** NK and NKT cell subsets within the CD45^{+} compartment in BM **(C)** and spleen **(D)**, analyzed by flow cytometry following treatment with clone 3H3. **(E-F)** Apoptosis analysis of NK cells in BM **(E)** and spleen **(F)** by flow cytometry using Annexin V and propidium iodide (PI) staining. **(G-H)** ΔMFI of Tim-3, Lag-3 and PD-1 in NK cells in BM **(G)** and spleen **(H)** after treatment. **(I-J)** CD16^{+} and CD16^{-} NK cell subsets within the CD45^{+} compartment in BM **(I)** and spleen **(J)**, analyzed by flow cytometry following treatment with clone 3H3. **(K-L)** ΔMFI of CD25 and CCR5^{+} migratory NK cell subset in BM **(K)** and spleen **(L)**, analyzed by flow cytometry following treatment with clone 3H3. n=5 per group. Statistical analysis was performed by Mann-Whitney U test: *$p<0.05$, **$p<0.01$, error bars indicate SD.

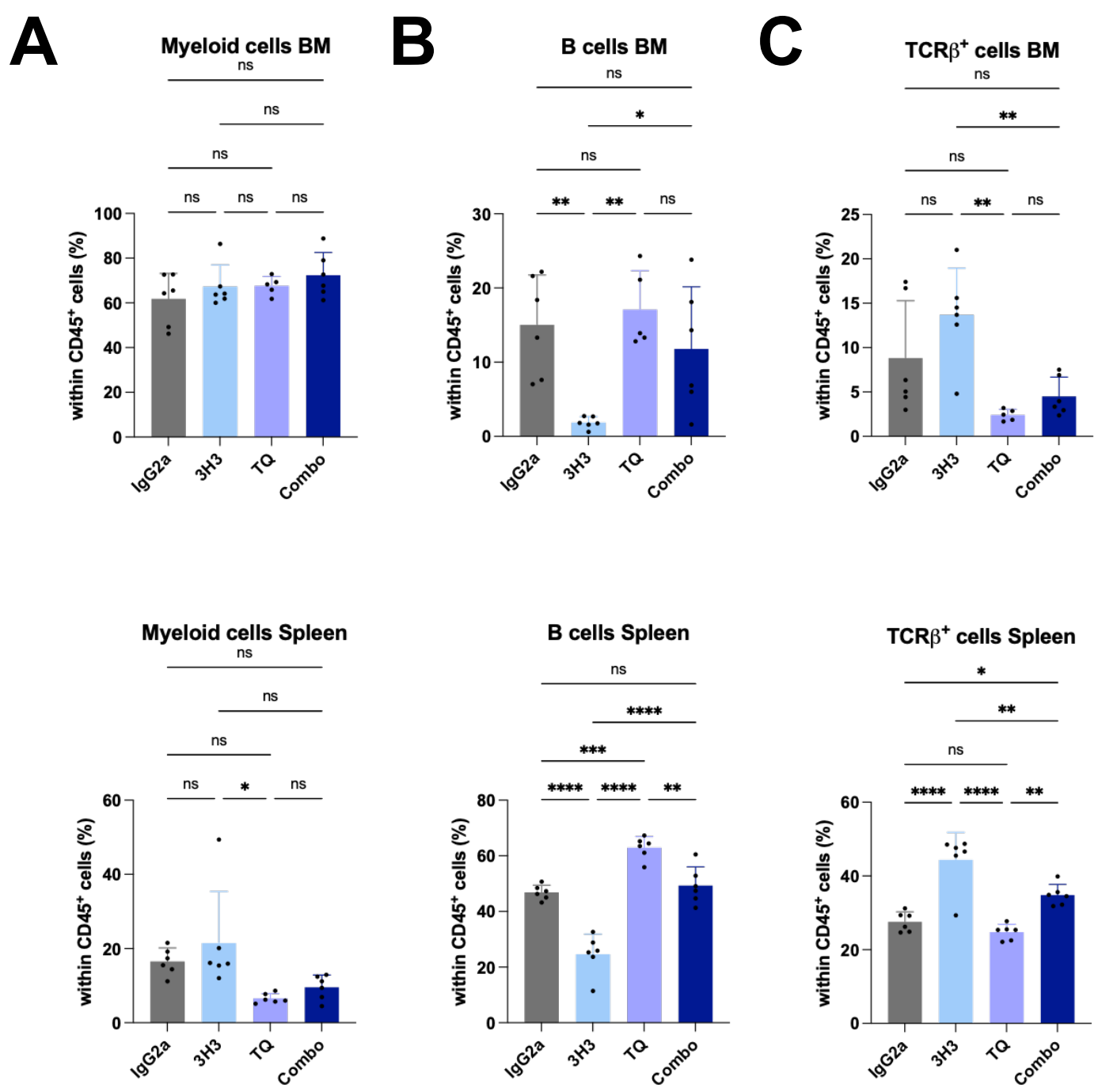

Suppl. Figure 4: Combination therapy with α4-1BB clone 3H3 and tasquinimod alters frequencies of immune subsets. (A-C) Bar graphs showing the frequencies of myeloid cells ($CD11b^+$) **(A)**, B cells ($CD19^+$) **(B)**, and T cells ($TCR\beta^+$) **(C)** within the $CD45^+$ compartment in BM and spleen, analyzed by flow cytometry. n=6 per group. Statistical analysis was performed by One-way ANOVA: ns: $p \geq 0.05$, $*p < 0.05$, $**p < 0.01$, $***p < 0.001$, and $****p < 0.0001$, error bars indicate SD.

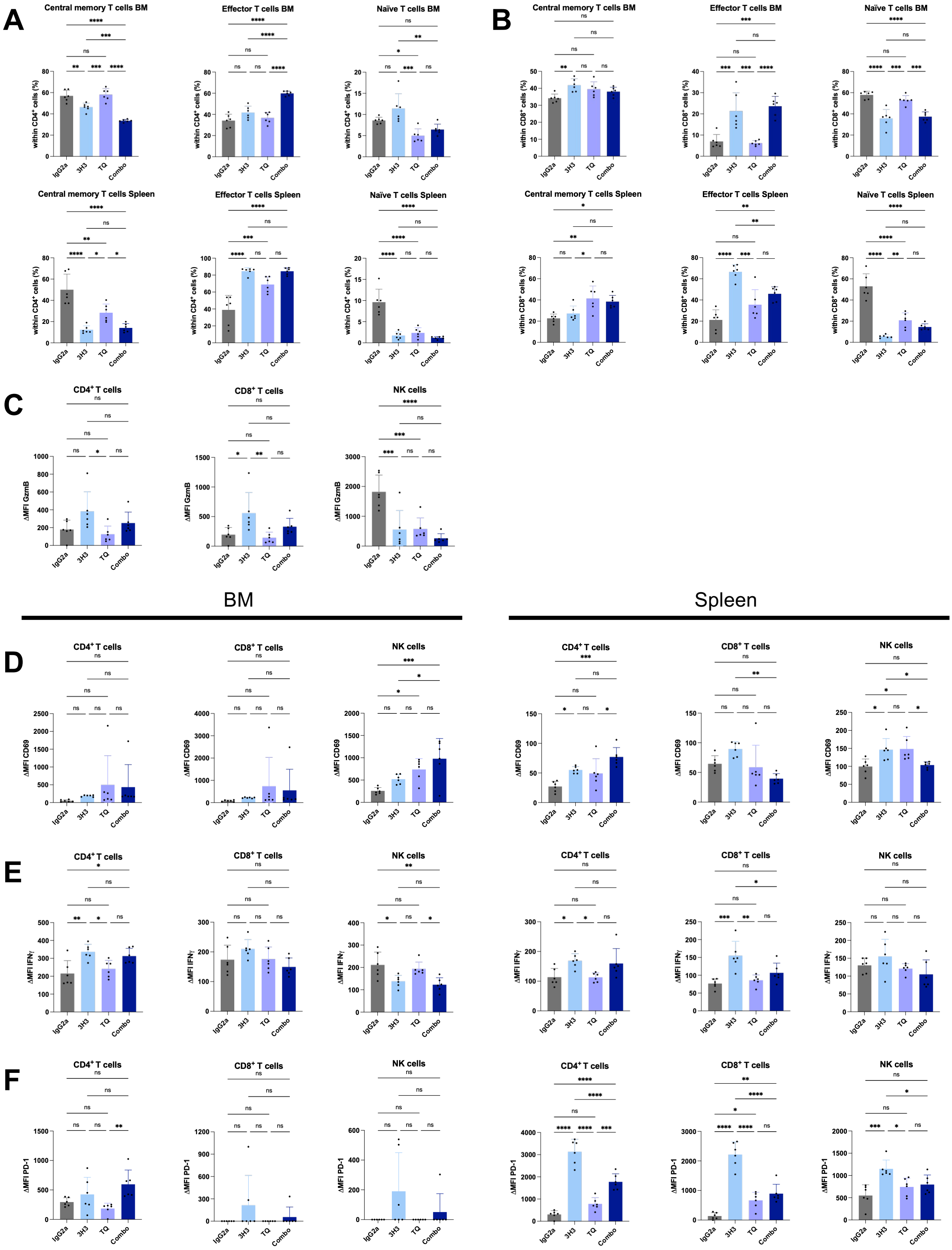

Suppl. Figure 5: Combination therapy induces Granzyme B–mediated T cell activation and effector differentiation. (A-B) Bar graphs show the distribution of CD4$^+$ **(A)** and CD8$^+$ **(B)** T cell subsets in BM and spleen, including central memory T cells (CD44$^+$CD62L$^+$), effector T cells

($CD44^+CD62L^-$) and naïve T cells ($CD44^-CD62L^+$) analyzed by flow cytometry. **(C)** ΔMFI of GzmB in lymphoid subsets from spleen. **(D-F)** ΔMFI of CD69 **(D)**, IFN-γ **(E)** and PD-1 **(F)** in lymphoid subsets from BM (left panel) and spleen (right panel). n=6 per group. Statistical analysis was performed by One-way ANOVA: ns: $p \geq 0.05$, *$p<0.05$, **$p<0.01$, ***$p<0.001$, and ****$p<0.0001$, error bars indicate SD.

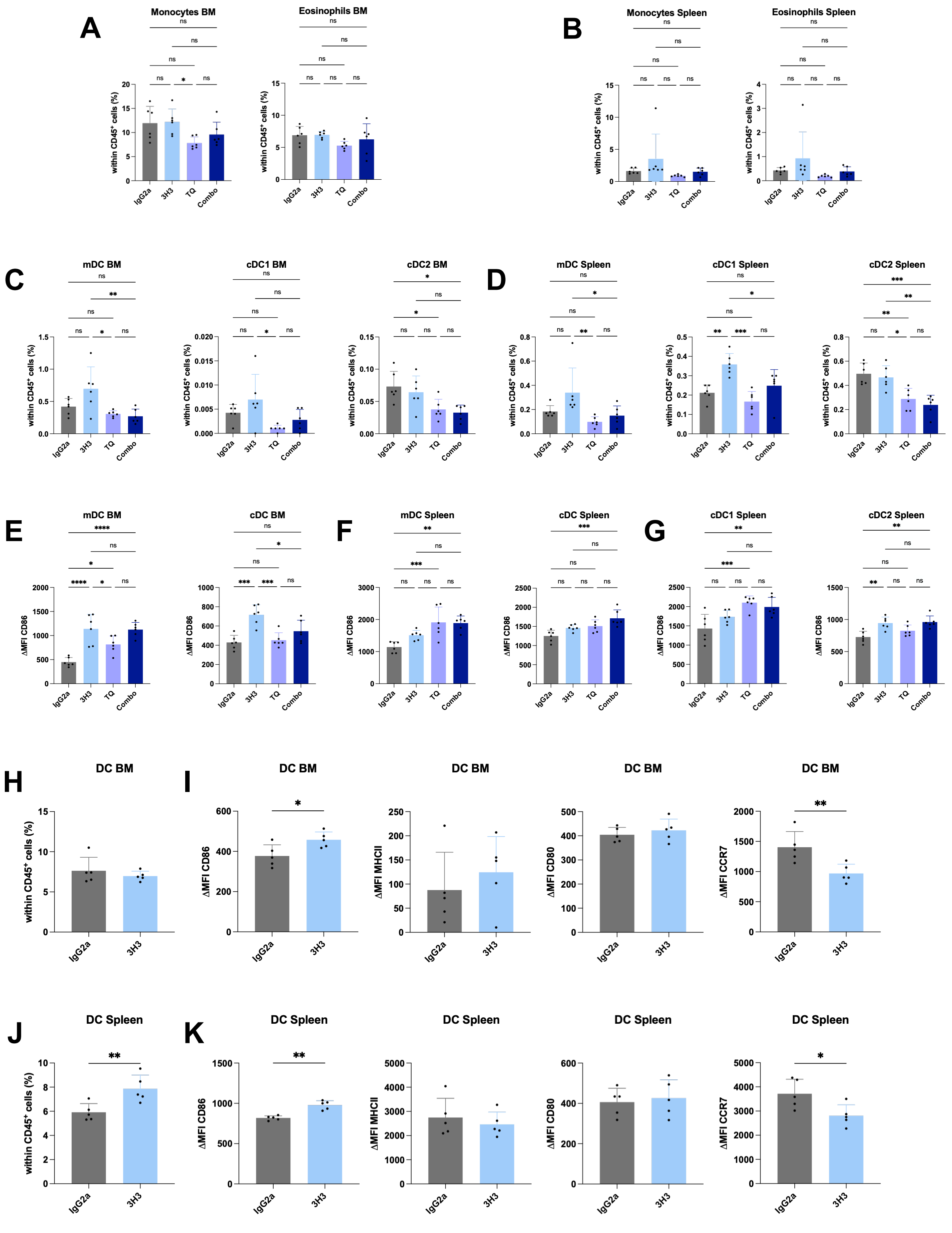

Suppl. Figure 6: Combination therapy orchestrates the reshaping of the tumor immune microenvironment. (A-B) Bar graphs showing the frequencies of myeloid subsets including monocytes (CD11b$^+$ Ly6G$^-$ Ly6C$^+$) and eosinophils (CD11b$^+$ Ly6G$^-$ Siglec-F$^+$) within the CD45$^+$ population in BM **(A)** and spleen **(B)**. **(C-D)** Bar graphs showing the frequencies of monocyte-

derived dendritic cells (CD11c$^+$ MHCII$^+$ CD64$^+$), conventional dendritic cell type 1 (cDC1: CD11c$^+$ MHCII$^+$ CD64$^+$ XCR1$^+$), and conventional dendritic cell type 2 (cDC2: CD11c$^+$ MHCII$^+$ CD64$^+$ CD11b$^+$) within the CD45$^+$ compartment in BM **(C)** and spleen **(D)**, analyzed by flow cytometry. **(E-F)** ΔMFI of CD86 in mDC and cDC from BM **(E)** and spleen **(F)**. **(G)** ΔMFI of CD86 in cDC1 and cDC2 from spleen. n=6 per group. Statistical analysis was performed by One-way ANOVA: ns: p≥0.05, *p<0.05, **p<0.01, ***p<0.001, and ****p<0.0001, error bars indicate SD. **(H-J)** Bar graphs showing the frequencies of dendritic cells in BM **(H)** and spleen **(J)** analyzed by flow cytometry following monotherapy with clone 3H3, sacrificed at 10 days post-inoculation. **(I-K)** ΔMFI of CD86, MHCII, CD80 and CCR7 in dendritic cells in BM **(I)** and spleen **(K)**. n=5 per group. Statistical analysis was performed by Mann-Whitney U test: *p<0.05, **p<0.01, error bars indicate SD.
