# Supplementary material for: IgG2a-formatted 4-1BB agonism combined with S100A9 inhibition enhances T cell activation and tumor control in a preclinical model of multiple myeloma
Source: J Exp Clin Cancer Res. 2026 May 4;45:172. doi: 10.1186/s13046-026-03716-4 (PMC13459263; doi:10.1186/s13046-026-03716-4)
Supplement: Supplementary file 2 [file 13046_2026_3716_MOESM1_ESM.docx]

| **Marker** | **Fluorochrome** | **Catalog #** | **Manufacturer** |
| --- | --- | --- | --- |
| **4-1BB** | PE | #106105 | Biolegend |
| **4-1BB Isotype ctrl** | PE | #402008 | Biolegend |
| **Annexin V** | APC | #550475 | BD Biosciences |
| **CCR5** | PE | #559923 | BD Biosciences |
| **CCR7** | BV605 | #120125 | BD Biosciences |
| **CD11b** | PECy7 | #101216 | Biolegend |
| **CD11c** | PerCP | #117326 | Biolegend |
| **CD16** | BV421 | #158029 | Biolegend |
| **CD19** | AF700 | #115528 | Biolegend |
| **CD19** | BV421 | #115538 | Biolegend |
| **CD25** | PECy7 | #101916 | Biolegend |
| **CD3** | AF700 | #100216 | Biolegend |
| **CD4** | BV605 | #100548 | Biolegend |
| **CD44** | BV421 | #103040 | Biolegend |
| **CD45** | APCCy7 | #103116 | Biolegend |
| **CD62L** | PE | #104408 | Biolegend |
| **CD64** | BV605 | #139323 | Biolegend |
| **CD64** | AF647 | #139322 | Biolegend |
| **CD80** | PE/Dazzle-594 | #104738 | Biolegend |
| **CD86** | PE/Dazzle-594 | #105042 | Biolegend |
| **CD8a** | PE/Dazzle-594 | #100762 | Biolegend |
| **CXCR2** | PE/Dazzle-594 | #149318 | Biolegend |
| **F4/80** | BV605 | #123133 | Biolegend |
| **Foxp3** | PE | #560408 | BD Biosciences |
| **Granzyme B** | AF647 | #515406 | Biolegend |
| **IFN-γ** | BV421 | #505830 | Biolegend |
| **Lag-3** | PE/Dazzle-594 | #125224 | Biolegend |
| **Ly-6C** | AF700 | #128024 | Biolegend |
| **Ly-6G** | AF700 | #127622 | Biolegend |
| **Ly-6G** | PerCPCy5 | #127616 | Biolegend |
| **MHC-II** | BV421 | #107632 | Biolegend |
| **NK-1.1** | PerCPCy5 | #108728 | Biolegend |
| **PD-1** | FITC | #135214 | Biolegend |
| **PD-L1** | PE/Dazzle-594 | #124324 | Biolegend |
| **PI** |  | #421301 | Biolegend |
| **Siglec F** | PE | #552126 | BD Biosciences |
| **TCR β** | AF700 | #109224 | Biolegend |
| **TCR γ/δ** | FITC | #118106 | Biolegend |
| **Tim-3** | BV421 | #119723 | Biolegend |
| **XCR1** | PE | #148204 | Biolegend |

**Supplementary Table 1:** Flow cytometry antibodies used in mouse experiments. Listing target marker, fluorochrome, catalog number and manufacturer.

| **Marker** | **Fluorochrome** | **Catalog #** | **Manufacturer** |
| --- | --- | --- | --- |
| **4-1BB** | BV421 | #309820 | Biolegend |
| **4-1BB Isotype ctrl** | BV421 | #400157 | Biolegend |
| **CD3** | APCCy7 | #317342 | Biolegend |
| **CD4** | BV605 | #300556 | Biolegend |
| **CD45** | PE/Dazzle-594 | #368530 | Biolegend |
| **CD56** | PE | #555516 | BD Biosciences |
| **CD8** | AF700 | #388808 | Biolegend |
| **Granzyme B** | PECy7 | #372214 | Biolegend |
| **IFN-γ** | PerCPCy5 | #502526 | Biolegend |

**Supplementary Table 2:** Flow cytometry antibodies used in human, *ex vivo* patient experiments. Listing target marker, fluorochrome, catalog number and manufacturer.
